# Reduced hysteresis in La_0.7_Ce_0.3_Fe_11.5_Si_1.5_ hydrides by grain size reduction

**DOI:** 10.1080/14686996.2025.2525742

**Published:** 2025-06-30

**Authors:** Mitali Madhusmita Prusty, Sri Harsha Molleti, Hiroto Takanobu, Sai Rama Krishna Malladi, Xin Tang, Hossein Sepehri-Amin

**Affiliations:** a Green Magnetic Material Group, Research Center for Magnetic and Spintronic Materials, National Institute for Materials Science, Tsukuba, Japan; b Department of Materials Science and Metallurgical Engineering, Indian Institute of Technology Hyderabad, Sangareddy, India; c Surface and Bulk Analysis Unit, Research Network and Facility Services Division, National Institute for Materials Science, Tsukuba, Japan

**Keywords:** Magnetocaloric materials, Ce-substitution, melt-spinning, room temperature magnetic refrigeration, La(Fe,Si)_13_ compound

## Abstract

Magnetic cooling technology, based on the magnetocaloric effect (MCE), offers an energy-efficient and eco-friendly alternative to conventional gas compression, but is often hindered by large magnetic hysteresis, which limits cyclic performance. In this study, we show that the hysteresis of La_0.7_Ce_0.3_(Fe,Si)₁₃ hydrides – a promising material for room-temperature refrigeration – can be significantly reduced by refining the microstructure of the precursor alloy. Substituting Ce for La in (La_0.7_Ce_0.3_)(Fe,Si)_13_H_x_ increases hysteresis losses from 12.3 J/kg to 34 J/kg. However, preparing the precursor alloy using the melt-spinning technique can almost eliminate this hysteresis. Lorentz transmission electron microscopy (Lorentz-TEM) shows that phase transition nucleation preferentially occurs at the grain boundaries. The hydrides prepared from melt-spun ribbons exhibit a much larger volume fraction of grain boundaries due to finer grains, providing a higher density of nucleation sites. This reduces the energy barrier for the phase transition and weakens the magneto-structural phase transition, as confirmed by *in-situ* X-ray diffraction patterns. Consequently, the reduced phase transition energy barrier leads to significantly lower hysteresis in melt-spun hydrides samples. These findings demonstrate the potential of microstructure engineering to reduce hysteresis in (La,Ce)(Fe,Si)_13_Hₓ materials for room-temperature magnetocaloric applications.

## IMPACT STATEMENT

Microstructure refinement via melt-spinning significantly reduces hysteresis in La_0.7_Ce_0.3_(FeSi)_13_ hydrides by reduction of energy barrier for phase transition, enhancing magnetocaloric performance for room-temperature applications.

## GRAPHICAL ABSTRACT

**Figure:**
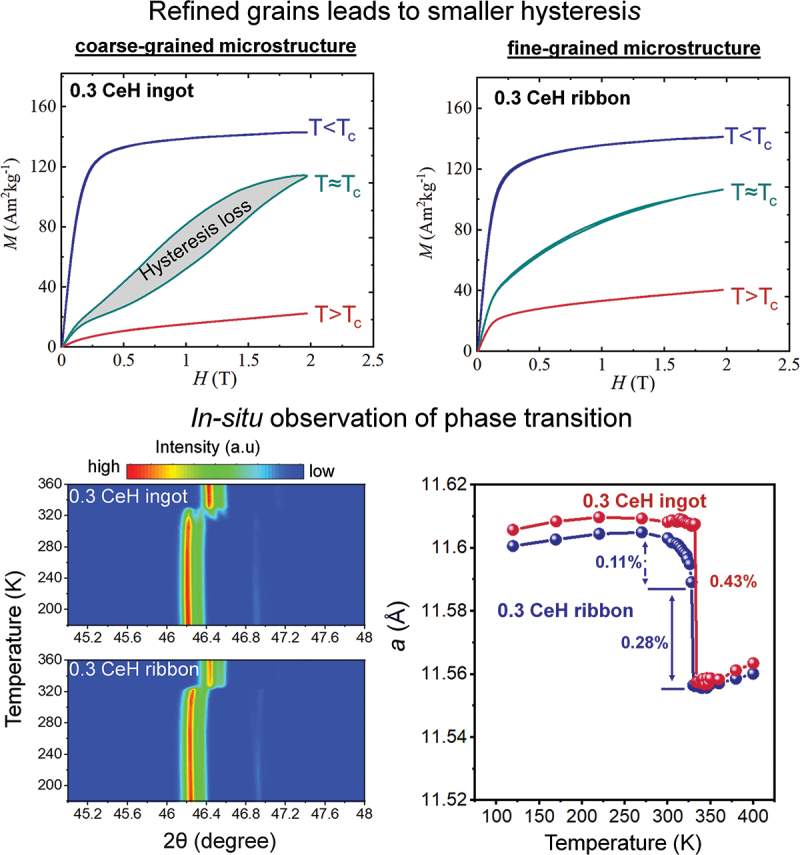

## 1. Introduction

Magnetic refrigeration utilizing the magnetocaloric effect (MCE) is a promising alternative to traditional vapor compression cooling technologies, displaying benefits in both environmental sustainability and energy efficiency [1]. This interest has driven extensive research on magnetocaloric materials. The discovery of Gd₅(Si_1-x_Geₓ)₄ [2] triggered a search for materials with significant MCE, such as rare earth free compounds, (Mn,Fe)_2_(P,As,Ge) [3,4], MnAs-based compounds [5], Mn_30_Fe_20-x_Cu_x_Al_50_ [6] and rare earth lean La(Fe_x_Si_1-x_)_13_ [7,8] that operate near room temperature.

Among these materials, La(Fe_x_Si_1-x_)_13_ (1:13 phase) compounds stand out as promising candidates due to their large magnetocaloric effect, which arises from a magneto-structural phase transition at the Curie temperature (*T*_C_ ≈ 203 K), where a paramagnetic/ferromagnetic phase transition takes place [9–12]. This phenomenon is driven by the itinerant behavior of 3d electrons in rare-earth and transition-metal-based compounds, known as the itinerant electron metamagnetic (IEM) transition, resulting in a large MCE [13,14]. In addition, Fe-rich La(Fe_x_Si_1-x_)_13_ compounds are considered excellent candidates for magnetic refrigeration due to their low cost, nontoxic constituents, large MCE, improved thermal conductivity by metal binder [14,15] and enhanced corrosion resistance [14,16]. Although the growing demand for gas liquefaction has spurred the development of cryogenic magnetic refrigeration materials [17], La(FeₓSi_1-x_)_13_-based compounds exhibit low MCE at cryogenic temperatures [18]. As a result, these materials are considered more suitable for applications near room temperature. However, several limitations – such as a low Curie temperature (*T*_C_ ≤ 210 K), significant magnetic hysteresis, and poor cyclic and mechanical stability – hinder their practical use in room temperature magnetic refrigeration systems [19–22].

To achieve large MCE near room temperature, extensive studies have focused on elemental doping. For instance, increasing Si concentration can raise the transition temperature to 222 K. However, this drives the transition to a second-order-like phase transition, reducing the MCE [12,23]. Substituting Co at the Fe site can increase *T*_C_ to near room temperature, but this approach reduces the IEM effect, thus results in a lower magnetic entropy change [23]. Meanwhile, introducing interstitial carbon atoms expands the lattice, raising *T*_C_ from 195 K at x = 0 to 250 K at x = 0.6 for LaFe_11.5_Si_1.5_C_x_ but also reduces ΔS_m_ [24,25]. Hydrogen insertion stands out among these dopants, effectively raising *T*_C_ above room temperature with minimal reduction in magnetic entropy [26]. For example, hydrogenation increase *T*_C_ to approximately 323 K, with a large magnetic entropy of 23 J/kg·K under a 5 T magnetic field [26]. To further enhance MCE in La(Fe,Si)_13_H_x_, studies have shown that partial substitution of Ce for La strengthens the IEM transition [27–29]. This effect originates from the change in band structure, which improves the magnetic entropy change [28–30]. However, this also results in an increased hysteresis, which is undesirable [31,32]. Cerium is an earth-abundant, cost-effective element, comprising up to 92–98% of rare earth content in many natural minerals [33]. Thus, developing a hysteresis-free Ce-containing La(Fe,Si)_13_H_x_ compound is beneficial for magnetic refrigeration.

In this study, we have focused on the La_0.7_Ce_0.3_(Fe,Si)_13_-based composition, examining how microstructure, specifically grain size and secondary phase distribution, affects the magnetocaloric effect. Melt spinning was chosen as the synthesis method due to its ability to produce rapidly solidified, homogeneous microstructures with fine grain size, enabling a systematic study of the impact of grain size on thermal and magnetic hysteresis. Compared to the conventional techniques such as arc melting or induction melting [34,35], melt spinning accelerates formation of the 1:13 phase and achieves the desired microstructure in the precursors with significantly shorter annealing time. As a result, this method led to a refined microstructure and substantially reduced hysteresis in (La,Ce)(Fe,Si)_13_H_x_ samples compared to those prepared via induction melting.

## 2. Experimental

Polycrystalline La_1-x_Ce_x_Fe_11.5_Si_1.5_ (x = 0, 0.3) alloys were prepared by induction melting under a high purity argon atmosphere, denoted as Ce0 ingot and Ce0.3 ingot, respectively. 5 wt.% of extra La and Ce was added in order to compensate evaporation of La and Ce during the melting process. The obtained ingots were polished to remove the oxidized surface. The ingots were wrapped in tantalum foils, sealed in quartz ampoules under an argon atmosphere, and annealed at 1373 K for five days and consequently quenched in water. The polished as cast ingots were used for melt spinning at an optimal speed of 30 m/s of the rotating copper wheel, the process was carried out in an argon atmosphere at a chamber pressure of 0.1 atm. An injection pressure of 3 kPa above the chamber pressure was applied to drive the melt through a fixed 0.8 mm diameter nozzle. The nozzle-to-wheel distance was maintained at 5 mm. These parameters were optimized to ensure the formation of high-quality rare-earth transition metal-based compounds [36]. The Ce0 and Ce0.3 as spun ribbons were then wrapped in tantalum foil and annealed in an argon atmosphere at 1373 K for 5 hours and subsequently followed by water quenching, which are referred as Ce0 ribbon and Ce0.3 ribbon, respectively. The annealed ingots and annealed ribbons were pulverized into powders smaller than 200 µm in size for hydrogenation. Hydrogenation was performed at 300 °C under a hydrogen gas flow at 1 atmospheric pressure for 5 hours [37]. The hydrogenated Ce-free and Ce-substituted samples prepared from ingot and melt-spun samples are denoted as Ce0H ingot, Ce0.3 H ingot, Ce0H ribbon and Ce0.3 H ribbon respectively. Powder X-ray diffraction (XRD, Rigaku Japan) has been performed using a 0.6 kW Rigaku Mini Flex diffractometer (Cr Kα radiation, λ = 2.2909 Å) at room temperature. Lattice parameters as well as phase fractions were calculated using Rietveld refinement of the XRD data (GSAS-II). The temperature-dependent XRD was performed on Rigaku SmartLab diffractometer using the Cu Kα radiation (λ = 1.5418 Å, operated at 45 kV and 200 mA) equipped with a temperature chamber (TTK 600, Anton Paar). Variable temperature XRD measurements were performed in a nitrogen atmosphere at 1 atmospheric pressure, with a heating process form the lowest temperature (120 K) to 400 K and the rate of 2 K/min. Once the target temperature was reached, the measurement was made after holding for 5 minutes for homogenization of the sample temperature. The differential scanning calorimetry (DSC, NETZSCH, Germany) data was obtained using NETZSCH DSC 200F3 from 273 K to 373 K at temperature rate of 5 K/min under an Ar flow condition (100 ml/min). The general microstructure was investigated using scanning electron microscopy (SEM, Carl Zeiss, Germany), Carl Zeiss Cross Beam 1540 ESB, equipped with energy dispersive X-ray spectroscopy (EDS) detector. Lorentz transmission electron microscopy (Lorentz-TEM, Thermofisher, USA) and detailed TEM observations were performed using a Titan G2 80–200. Atom probe tomography (APT, CAMECA, USA) samples were prepared using the lift-out technique on a G4-UX dual-beam system. APT experiments were performed with a laser-assisted local electrode atom probe (CAMECA LEAP 5000 XS) operated in laser mode at a specimen temperature of 50 K, with a laser pulse energy of 30 pJ and pulse rate of 250 kHz. Magnetic measurements were conducted by a superconducting quantum interference device vibrating sample magnetometer, SQUID-VSM (MPMS SQUID VSM, Quantum Design, USA). The magnetic hysteresis loops were measured at the transition temperature by first increasing the magnetic field to 2 T, followed by decreasing it to 0 T at a rate of 300 Oe/s. To ensure accurate and reproducible measurements, we adopted the ‘loop method’ [38]: the sample was first heated well above the transition temperature to eliminate any thermal or magnetic history effects. Subsequently, the temperature was lowered to the target measurement temperature before recording the hysteresis loop. This procedure minimizes the influence of prior measurements and ensures that the system reaches thermal equilibrium before data collection.

## 3. Results and discussion

### 3.1. Structural analysis of non-hydrogenated and hydrogenated samples

**Figure 1.**
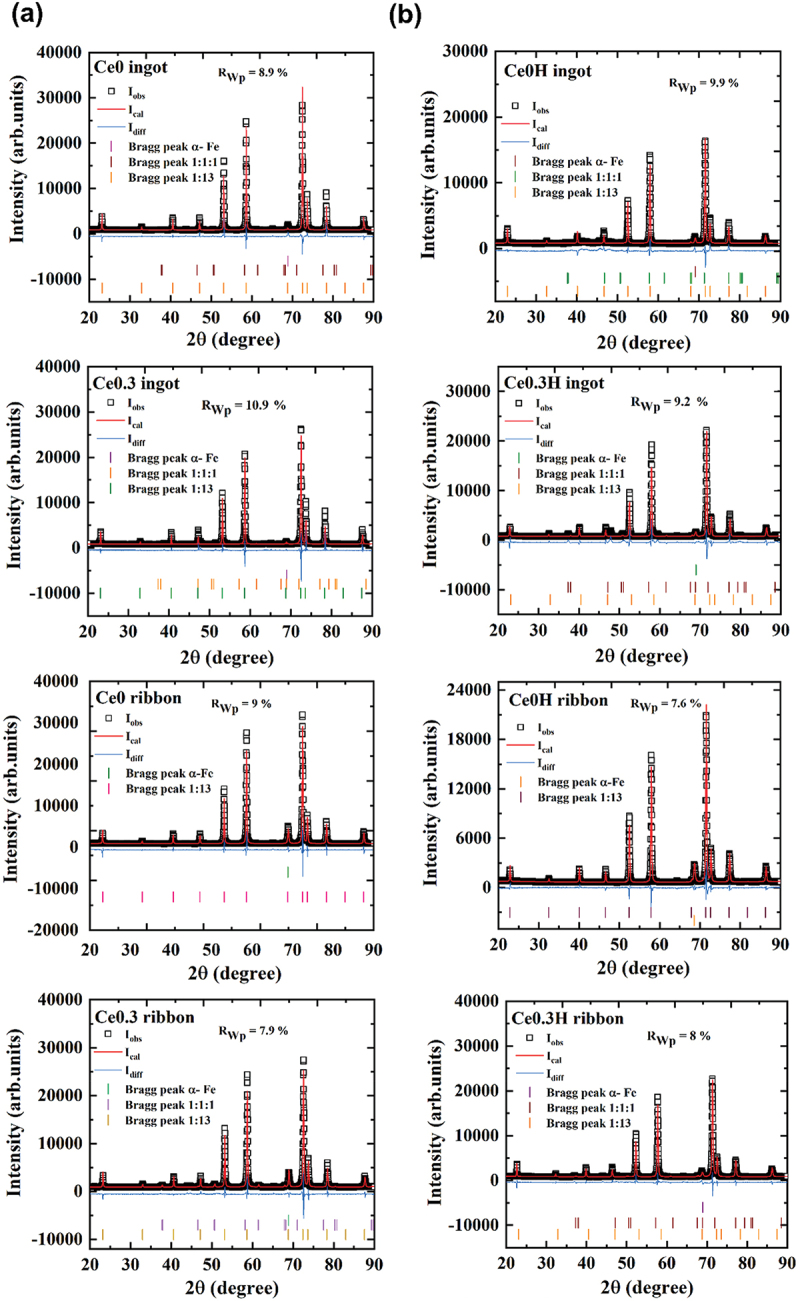
Room temperature XRD pattern and Rietveld refinement of (a) Ce0 ingot, Ce0.3 ingot, Ce0 ribbon, Ce0.3 ribbon, (b) their corresponding hybrids.

Room temperature X-ray diffraction (XRD) data for optimally annealed samples of induction-melted Ce0, Ce0.3, melt-spun Ce0 and Ce0.3 ribbons are shown in Figure 1(a). After annealing, all samples crystallized in the NaZn_13_-type cubic structure (space group: Fm$\overset{ˉ}{3}$c), known as the 1:13 phase. Along with this desired 1:13 phase, a minor amount of *α*-Fe impurity was observed in all samples. In addition, the LaFeSi (1:1:1) minor phase appeared in Ce-substituted ingot and melt-spun samples. Rietveld refinement, summarized in Table 1, confirms that each sample contains over 92 wt.% of the 1:13 phase. Interestingly, *α*-Fe impurities decreased with partial substitution of La by Ce in the ingot sample. It is important to note that the presence of secondary phases can alter the Fe:Si ratio within the 1:13 matrix, which in turn affects both the lattice constant and the transition temperature. An increase in the Fe:Si ratio in LaFe_13_-ₓSiₓ generally leads to an expansion of the unit cell, thereby increasing the lattice constant and reducing the Curie temperature [39,40]. All annealed ingots and ribbons were subsequently subjected to hydrogenation. Room-temperature X-ray diffraction (XRD) data for the hydrogenated Ce0H and Ce0.3 H ingots, and Ce0H and Ce0.3 H melt-spun ribbons are presented in Figure 1(b). The data confirms that the main phase in all samples remains the same as non-hydrogenated samples, NaZn_13_-type cubic structure with some minor phases of α-Fe and 1:1:1. In contrast to the non-hydrogenated samples, the 1:13 phase diffraction peaks of the hydrides shift to lower angles, indicating lattice expansion due to hydrogen atom insertion [24,41]. The atomic positions and occupancies based on refinement, and refinement parameters are provided in Supplementary tables S1–S3.

**Table 1.** List of lattice parameters of NaZn_13_ type phase, *T*_C_, weight fraction of 1:13, α-Fe and 1:1:1 phases of non-hydrogenated samples.

Sample	a (Å)	T_C_ (K)	1:13 (wt.%)	α-Fe (wt.%)	1:1:1 (wt.%)
Ce0 ingot	11.476(8)	195	94.4	5.6	0
Ce0 ribbon	11.484(5)	199	93.2	6.8	0
Ce0.3 ingot	11.454(1)	169	98.9	0.6	0.5
Ce0.3 ribbon	11.460(2)	176	92.2	5.3	2.5

**Table 2.** List of lattice parameters of NaZn_13_ type phase, *T*_C_, weight fraction of ferromagnetic and paramagnetic 1:13, α-Fe and 1:1:1 phase of hydrides.

Sample	a (Å)	*T*_C_ (K)	1:13 (wt%)	α-Fe (wt%)	1:1:1 (wt%)
Ce0H ingot	11.619(0)	336	96.2	2.9	0.9
Ce0H ribbon	11.605(5)	325	93.4	6.6	0
Ce0.3 H ingot	11.630(5)	332	95.9	2.2	1.9
Ce0.3 H ribbon	11.652(7)	328	94.9	3.2	1.9

### 3.2. Magnetic properties of non-hydrogenated and hydrogenated samples

**Figure 2.**
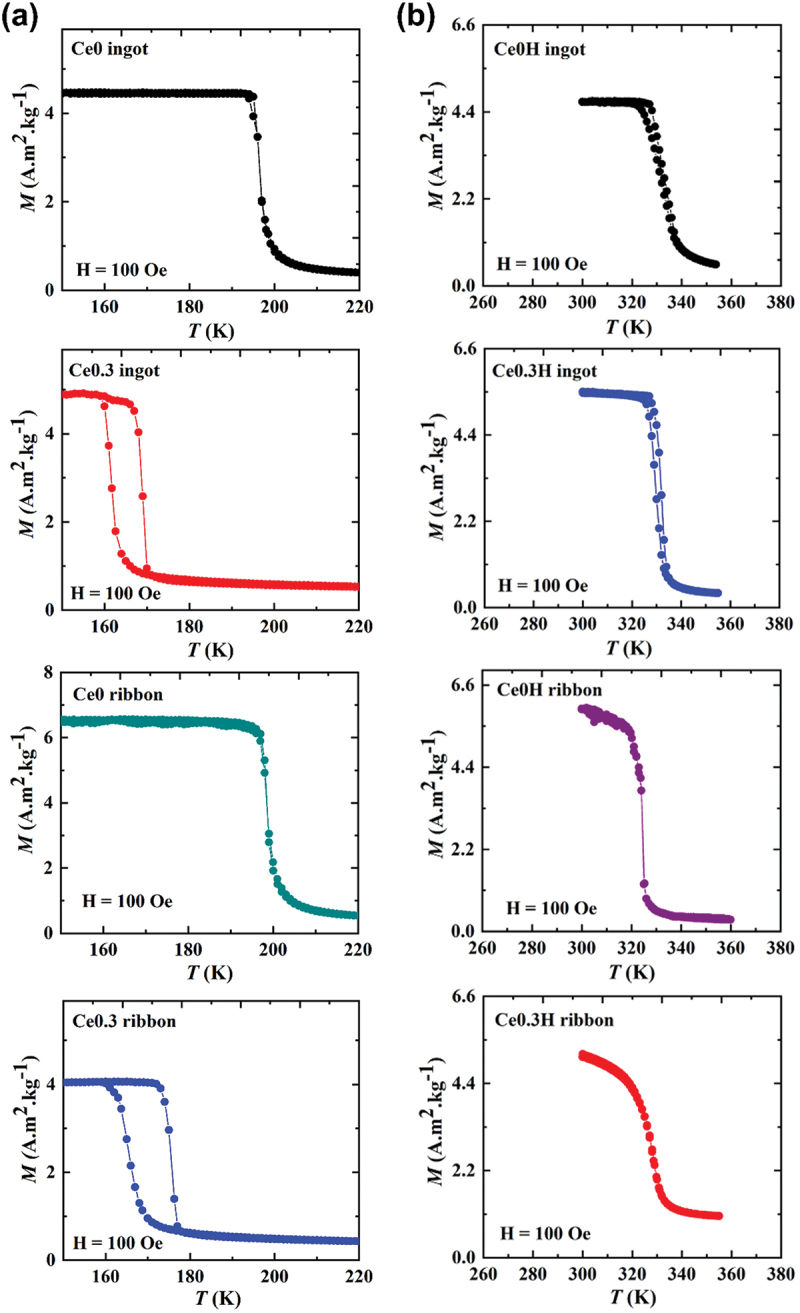
(a) Temperature dependence of the magnetization of Ce0 ingot, Ce0.3 ingot, Ce0 ribbon and Ce0.3 ribbon measured in a magnetic field of 0.01 T, (b) and their corresponding hydrides.

Curie temperatures (*T*_C_) for each sample, measured from *M-T* curves at 0.01 T are shown in Figure 2(a) and listed in Table 1. *T*_C_ was determined from the peak minima in *dM/dT* vs. *T* plots on the heating branch of *M-T* curves. A high-angle shift in the 1:13 diffraction peaks in Ce-substituted samples indicates lattice contraction, attributed to Ce substitution as shown in Table 1. This substitution reduces lattice constant, weakening Fe–Fe interactions and thus reduces *T*_C_ [42–44]. Moreover, thermal hysteresis increased significantly with Ce substitution, from 1.1 K in Ce0 ingots to 7.2 K in Ce0.3 ingots and 0.4 K in Ce0 ribbon to 10.4 K in Ce0.3 ribbons, consistent with the previous reports on Ce-substituted La(FeSi)_13_ compounds [18,45]. The thermal hysteresis observed in the Ce-substituted melt-spun ribbons is larger than that of the ingot samples, likely due to the combined effects of Ce doping and microstructural factors that strengthen the first-order transition characteristics [34,35], which implies the complex relations between chemical composition, processing methods, and hysteresis behaviour in these alloys.

Temperature-dependent magnetization measurements for Ce0H, Ce0.3 H ingots, Ce0H and Ce0.3 H melt-spun ribbons under a 0.01 T magnetic field, conducted during heating and cooling at 2 K/min, are shown in Figure 2(b). Hydrogenation led to an increase in *T*_C_ from 169 K to 332 K for the Ce0.3 ingot and from 176 K to 328 K for the Ce0.3 ribbon, as seen in Figure 2(a–b) and Table 2. Hydrogenation increased *T*_C_ to near room temperature while significantly reducing thermal hysteresis, as seen in Figure 2(b) and Table 3. The insertion of interstitial hydrogen expands the lattice, lengthening Fe–Fe bonds, which causes a stronger Fe–Fe exchange interaction and raises *T*_C_ [46,47]. It should be noted that the *T*_C_ shifts to higher temperature with higher fields shown in Supplementary Figure. S1, for example, in Ce0.3ribbon and Ce0.3 H ribbon samples, it is clearly observed for both samples that the *T*_C_ shift with magnetic field at a same rate of 4 K/T, and hydrogen absorption does not change its behaviour.

**Table 3.** List of Curie temperature (*T*_C_), thermal hysteresis (Δ*T*_hys_), Δ*T*_FWHM_, magnetic entropy change ($\Delta S_{m-2T}^{max}$) and relative cooling power (RCP) of Ce0H, Ce0.3 H ingots and Ce0H, Ce0.3 H melt-spun ribbons.

Sample	*T*_C_ (K)	Δ*T*_hys_ (K)	*MH*_L_ (J/kg)	δ*T*_FWHM_ (K)	${\Delta \;S}_{m}^{max}$ (J/kg K) ΔH = 2 T	RCP (J/kg)	Reference
Ce0H ingot	334	2.2	12.3	13.2	14.6	193.8	This work
Ce0H ribbon	329	0.3	1.7	11	16.7	184.2	This work
Ce0.3 H ingot	335	2.1	34	7.4	26.6	197.8	This work
Ce0.3 H ribbon	332	0.1	1.7	12.4	11	137	This work
La_0.6_Ce_0.4_Fe_11_Si_2_	–	–	20	–	12	–	[25]
(La_0.6_Ce_0.4_)_2_Fe_11_SiH_y_	313	0.5	0.6	–	12.2	–	[44]
LaFe_11.4_Si_1.6_H_0.86_	281.7	–	–	18	11.1	119.1	[39]
LaFe_11.7_Si_1.3_C_0.2_H_x_	308.5	–	2	–	14.4	–	[52]
La_0.5_Pr_0.5_Fe_11.4_Si_1.6_H_0.9_	260	1.4	11.7	–	22	–	[53]
La_0.5_Pr_0.5_Fe_11.4_Si_1.6_H_1.6_	317	0	2.3	–	17.7	–	[53]

In hydrides, the phase transition appears less sharp than in the parent compounds (Figure 2(a)), indicative of weakening of the IEM [26,41]. Of interest, the addition of Ce in the ingot precursor increases the thermal hysteresis due to the strengthening characteristic of first-order phase transition, which can also be evidenced in the sharper *M-T* curve near the *T*_C_. With preparing sample from melt-spun technique, we can observe the absence of thermal hysteresis along with reduced sharpness of the *M-T* curve for Ce0.3 H ribbon. The higher *T*_*C*_ values observed in our hydride samples than those reported in previous studies, likely due to near-saturation hydrogen absorption.

### 3.3. Magnetocaloric performance of non-hydrogenated and hydrogenated samples

Magnetic entropy change (Δ*S*_m_) was calculated from *M-T* curves of the heating branch measured under an applied magnetic field ranging from 0 to 2 T at a field step of 0.5 T using the Maxwell relation, which is expressed as

(1) $${\left(\frac{\partial S}{\mathrm{\partial H}}\right)}_{T}\;=\mu_{0}{\left(\frac{\mathrm{\partial M}}{\mathrm{\partial T}}\right)}_{H}$$

Where S is the total entropy, H is the magnetic field, M is the magnetization and T is the temperature. This relation provides a fundamental framework to relate changes in entropy and magnetization under varying magnetic fields and temperatures [48], using following equation.

(2) $$\Delta S_{m}=\mu_{0}\int_{H_{f}}^{H_{i}}\left(\frac{\partial M}{\mathrm{\partial T}}\right)\mathrm{dH}$$

Here, *H*_*i*_ and *H*_*f*_ are the initial and final values of applied magnetic field and μ_0_ is the permeability of free space [48].

**Figure 3.**
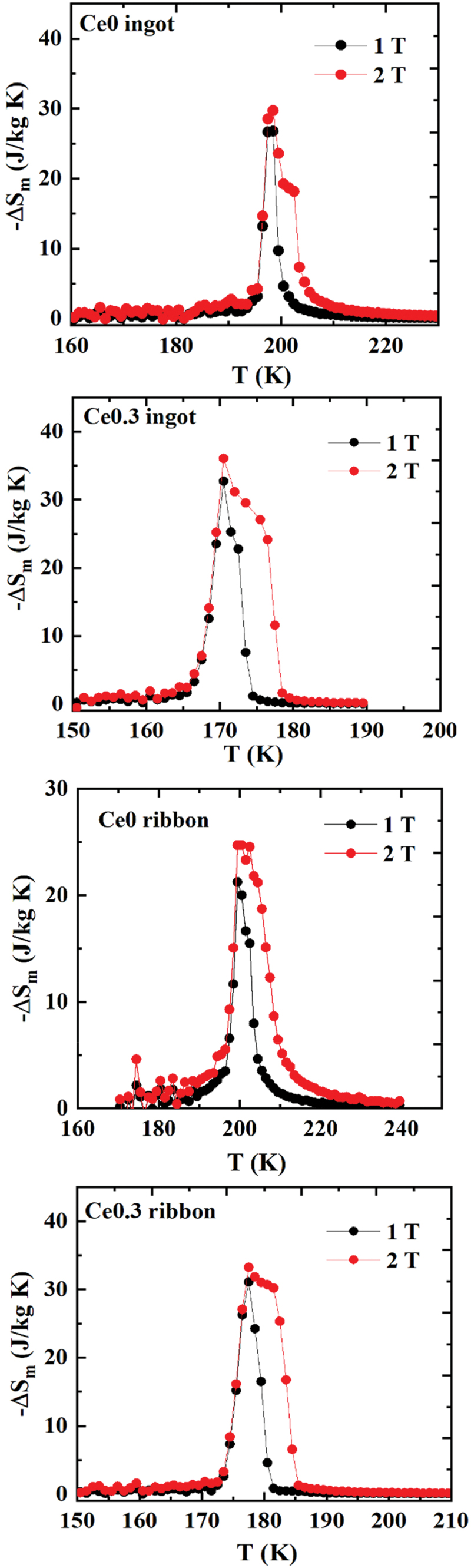
Temperature dependent ΔS_m_ of Ce0 ingot, Ce0.3 ingot, Ce0 ribbon and Ce0.3 ribbon under a maximum applied field of 2 T.

The maximum magnetic entropy change ($-\Delta S_{m}^{max}$) increased from 29.7 J/kg·K in the Ce-free ingot to 32.7 J/kg·K in the Ce0.3 ingot, while it was 24.7 J/kg·K in Ce-free ribbon and increased to 33.2 J/kg·K in the Ce0.3 ribbon under a 2 T field, shown in Figure 3. These values agree with previous reports on a similar composition [27,33,49,50], and the increase in the magnetic entropy change indicates a strengthening of the IEM in Ce-substituted samples [29,45,51]. It should be noted that the preparation method has minimal influence on magnetocaloric response as both ingot and melt-spun Ce0.3 samples show strong first-order phase transition and almost identical magnetic entropy change. Moreover, at an external field of 2 T, an asymmetric broadening of peak features in the ΔS_m_ vs T curves was observed. This can be attributed to the field-induced itinerant electron metamagnetic (IEM) transition [8], implying the complex relationship between magnetic ordering and external fields.

**Figure 4.**
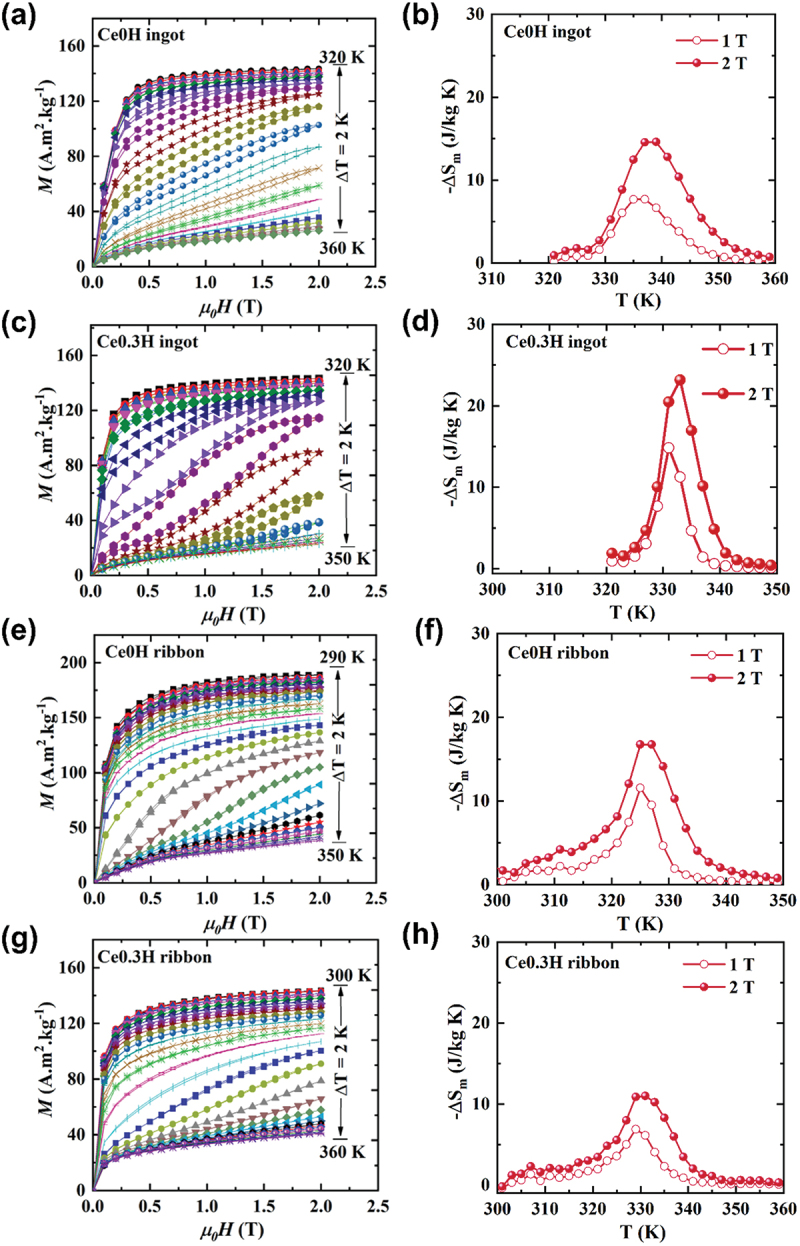
(a), (c), (e), and (g) Magnetization isotherms of Ce0H ingot, Ce0.3 H ingot, Ce0H ribbon, and Ce0.3 H ribbon measured near their respective *T*_C_. (b), (d), (f), and (h) Temperature-dependent entropy change (ΔS_*m*_) under a maximum applied field of 2 T for the corresponding samples.

The isothermal magnetization of Ce0H and Ce0.3 H ingots, and Ce0.3 H and Ce0H ribbons and their corresponding magnetic entropy change (Δ*S*_m_) under a maximum magnetic field of 2 T near *T*_C_ are shown in Figure 4(a–h), respectively. It should be noted that the isothermal magnetization was taken using the loop process to avoid the influence of hysteresis [38] and this method achieves same magnetic entropy change determined from *M-T* curves as shown in Supplementary Figure. S2.

In the hydrides, since the thermal hysteresis for all hydride samples is comparable, below 1 K. Herein, we use magnetic hysteresis loss (*MH*_L_) to evaluate the hysteresis, which is defined as the enclosed area between the ascending and descending branches of the M–H curves at the transition temperature. Magnetic hysteresis losses were found to be 12.3 J/kg, 34 J/kg, 1.7 J/kg, and 1.7 J/kg for the Ce0H and Ce0.3 H ingots, and Ce0H and Ce0.3 H ribbons, respectively. Notably, the ribbons exhibit lower magnetic hysteresis than ingot samples, suggesting that microstructural factors – particularly grain size reduction, which increases grain boundary area and increases internal strain – may play a role in hysteresis reduction [45]. The – $\Delta S_{m}^{max}$value increased substantially from 14.6 J/kg·K for the Ce0H ingot to 23 J/kg·K for the Ce0.3 H ingot under a 2 T field change. This increase could be attributed to strengthened first-order phase transition characteristics in Ce-substituted hydrides. Relative Cooling Power (RCP) is defined as the amount of heat transferred by a unit mass of refrigerant between the hot and cold reservoir during one refrigeration cycle. It can be expressed as the multiplication of the maximum value of Δ*S*_m_ and the full width half maxima of Δ*S*_m_ vs T curve [52].

$$\text{RCP}=\left|\text{Δ}S_{m}^{max}\right|x\text{ δ ​}{\text{T}}_{\text{FWHM}}$$

RCP serves as a critical measure of the usefulness of a magnetic refrigerant, as it quantifies the material’s capacity to transfer heat effectively over a temperature range, thereby indicating its practical applicability in magnetic refrigeration systems. RCP values for studied compound are summarized in Table 3. Ce0.3 H ingot exhibits the highest RCP value, while melt-spun Ce0.3 H ribbon displays the low RCP among all the samples. This reduction in RCP for the ribbon is attributed to a decrease in $\left|\Delta S_{m}^{max}\right|$.However, the broadening of -Δ*S*_*m*_ versus T curve for Ce0.3 H ribbon results in an increased δT_FWHM_, enabling its application across a wider range of temperature. The gradual transition in Ce0.3 H ribbon sample causes a lower (∂M(T, H))/∂T slope at the paramagnetic-to-ferromagnetic transition, resulting in a smaller $-\Delta S_{m}^{max}$ value and a broader -Δ*S*_m_ vs T curve, but with almost zero magnetic and thermal hysteresis. We also compared the properties achieved in this work with those reported in the literature, shown in Table 3. Our hydride samples demonstrate improvements in both $-\Delta S_{m}^{max}$ and RCP values, with comparable magnetic hysteresis to the previously reported data [30,41,45,53,54] as shown in Figure. S3 in Supplementary file. In addition, Ce0.3 H ribbon sample demonstrates the excellent stability and reproducibility over 10 cycles (See the DSC data in the Supplementary Figure. S4). For example, on heating, the latent heat (the area underneath of peak of DSC data) averages −6.59 J/g. The small deviation in latent heat across both heating and cooling cycles confirms that the material maintains reproducible thermodynamic properties, supporting its reliability for cyclic refrigeration applications.

### 3.4. Microstructure of non-hydrogenated and hydrogenated samples

**Figure 5.**
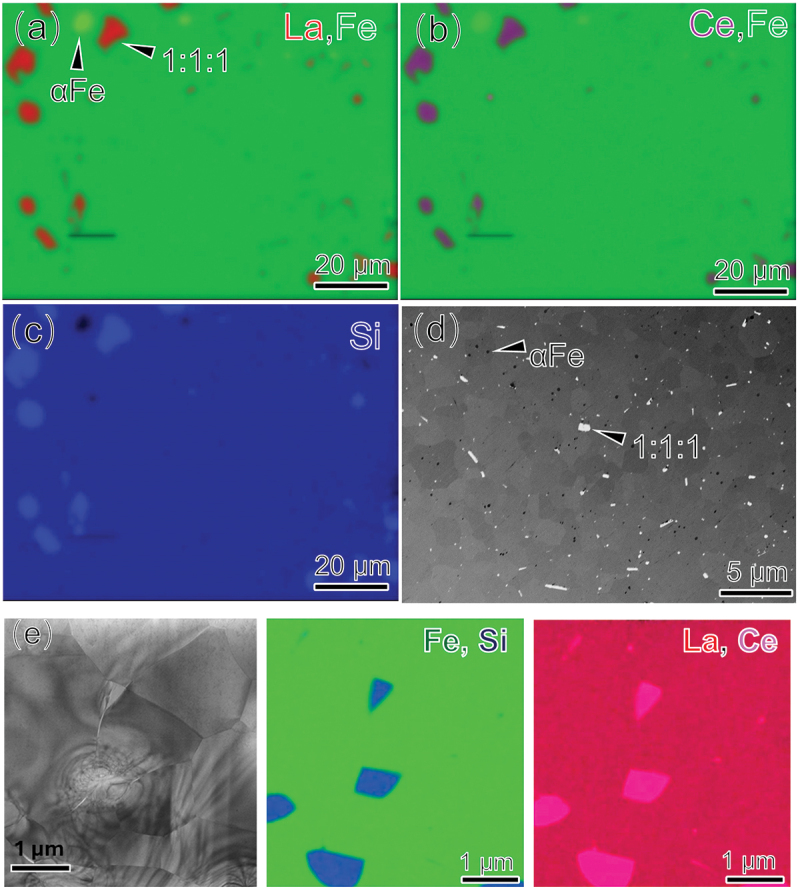
SEM-EDS maps of (a–c) annealed Ce0.3 ingots, (d) backscattered electron SEM image obtained from wheel surface of ribbon, and (e) HAADF-STEM image and elemental distribution of the annealed Ce0.3 ribbon.

**Figure 6.**
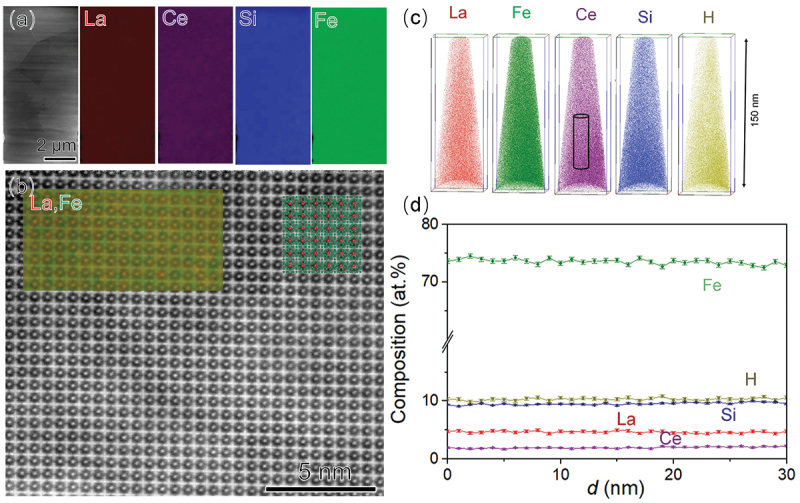
(a) HAADF-STEM image, EDS analysis, and (b) high-resolution HAADF-STEM image of the Ce0.3 H ribbon sample. The inset shows the atomic-resolution map of La and Fe elements and simulated structure, red and green spheres stand for La atoms and Fe+Si atoms, respectively; (c) the elemental maps of the matrix phase in Ce0.3 H ribbon obtained from atom probe tomography; (d) compositional profile determined from the selected black cylinder box.

The comparison of magnetic properties indicates that the hydride samples prepared using the melt-spun technique exhibit significantly lower magnetic hysteresis loss than the ingot samples, regardless of the composition. This difference is likely due to variations in microstructure. To explore this, we studied the microstructure of the Ce-containing (Ce0.3) ingot and melt-spun samples. Figure 5(a–c) show SEM-EDS maps of La, Ce, Fe, and Si for the Ce0.3 ingot, indicating in addition to the primary 1:13 phase, Si-rich 1:1:1 phase and α-Fe phase are present in the microstructure, with size ranging from 3 to 10 μm. Melt-spinning retained these phase constituents, as confirmed by the XRD data Figure 1(a). However, in melt-spun Ce0.3 ribbons, the 1:1:1 phase (bright contrast) and α-Fe phase (dark contrast) became more uniformly distributed, as shown in Figure 5(d). The average grain size of the melt-spun Ce0.3 ribbon was reduced to approximately 2–5 μm (Supplementary figure. S5), significantly smaller than the 15 μm grain size observed in the ingots [55,56]. TEM analysis (Figure 5(e)) confirmed the presence of the main secondary phase (1:1:1 phase) in Ce0.3 ribbon. Despite the existence of secondary phases, the composition of the 1:13 matrix phase remains nearly identical in both the Ce0.3 ingot and Ce0.3 ribbon samples as shown in Supplementary Figure. S6. This suggests that the melt-spinning process has a negligible influence on the composition of the matrix phase. The microstructure of the Ce0.3 ribbon hydride sample, shown in Figure 6(a–b)), reveals grains of 2–5 μm with a homogeneous distribution and no elemental segregation at grain boundaries. High-resolution HAADF-STEM imaging along the [001] direction shows highly ordered atomic arrangements, indicating that the crystal lattice remains undistorted after hydrogen absorption, with no observable nano-defects in the matrix phase. This contrasts with the nano crystallization observed in Ce0.4 H reported by Liu et al. [15]. To further investigate the microstructure, atom probe tomography (APT) was performed on this sample, as shown in the inset of Figure 6(c). The APT analysis reveals a uniform distribution of all constituent elements throughout the sample. The measured atomic composition is: Fe–73.45 at.%, Ce–2.11 at.%, Si–9.82 at.%, H–10.21 at.%, and La–4.42 at.% in this sample. This corresponds to an approximate hydride formula of (LaCe)(FeSi)_13_H_1.54_. The hydrogen content is consistent with previous report [57], which showed similar hydrogen concentrations and nearly identical Curie temperatures. Overall, the microstructural analysis reveals refined grain sizes and a more uniform distribution of secondary phases (1:1:1 and α-Fe) in the melt-spun samples.

### 3.5. Phase transition behaviours

**Figure 7.**
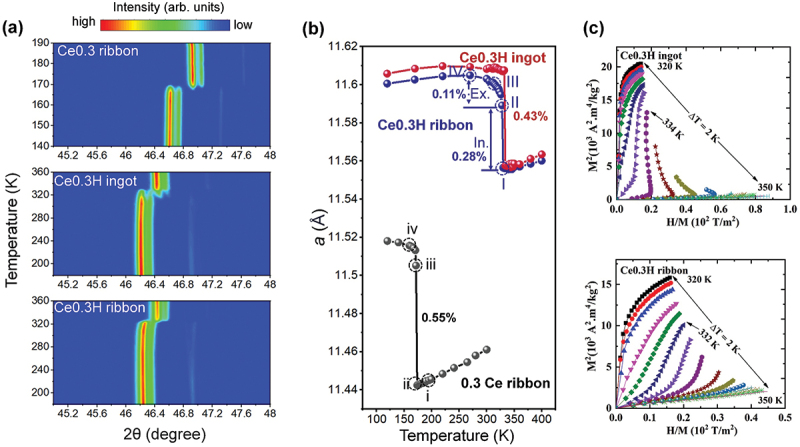
Temperature-dependent (a) XRD patterns and (b) lattice constants of Ce0.3 ribbon, Ce0.3 H ingot and Ce0.3 H ribbon, (c) Arrott plots of Ce0.3 H ingot and Ce0.3 H ribbon.

As shown in Figures 2–4, the microstructure in the non-hydrogenated state has a negligible influence on thermomagnetic behavior and magnetocaloric performance. However, a significant change was observed after hydrogenation. Therefore, we selected the Ce_0.3_ ribbon sample as a reference to investigate the differences in phase transition between the non-hydrogenated and hydrogenated states. Additionally, we explored how microstructural variations influence the phase transition by tracking phase evolution in hydride samples prepared from both ingots and melt-spun ribbons. Figure 7(a–b) present the temperature-dependent XRD patterns, showing a shift of the 1:13 peaks toward higher angles as the temperature increases across the transition temperatures of the three studied samples. This shift indicates a contraction of the lattice constant and a reduction in unit cell volume during the itinerant electron metamagnetic (IEM) transition. To further investigate the structural changes, the XRD data were refined in detail. Unlike previous studies [15], we recorded the XRD data with a finer step size of approximately 2 K, allowing more precise tracking of the phase transition. For the Ce0.3 ribbon sample, a 0.55% shrinkage in the lattice constant was observed. After hydrogenation, the phase transition occurred in two distinct steps: 1, A continuous reduction of the lattice constant as the temperature decreased from 300 K to 328 K, exhibiting characteristics of a gradual change in the lattice constant. This behaviour could be attributed to a weakened magneto-structural phase transition. 2, A sudden reduction in the lattice constant with a further increase in temperature to 300 K, indicative of a typical strong magneto-structural phase transition. A similar behaviour was reported by Lai et al. [20]. In contrast, for the Ce0.3 H ingot sample, the lattice constant remained stable until a sudden reduction of 0.43% occurred at the transition temperature. The temperature-dependent behaviour of the lattice constant between these two samples aligns with the *M-T* curves measured under a 100 Oe field, as shown in Figure 2. These factors result in a smaller sudden change in the lattice constant, inducing weakened first-order phase transition behaviour in the Ce0.3 H ribbon sample, which is reflected in the Arrott plot shown in Figure 7(c). The curvature of the Arrott plot serves as an indicator of the strength of the first-order phase transition, revealing a stronger first-order phase transition in the Ce0.3 H ingot sample. In contrast, the Ce0.3 H ribbon sample exhibits a positive slope in the Arrott plot, indicative of a weakened first-order phase transition [58], which is confirmed by the critical exponent analysis (shown in Supplementary Figure. S7) proposed by Law et al. [59]. As a result, the magnetic hysteresis loss is significantly reduced from 34 J/kg for the Ce0.3 H ingot to 1.7 J/kg for the Ce0.3 H ribbon sample.

**Figure 8.**
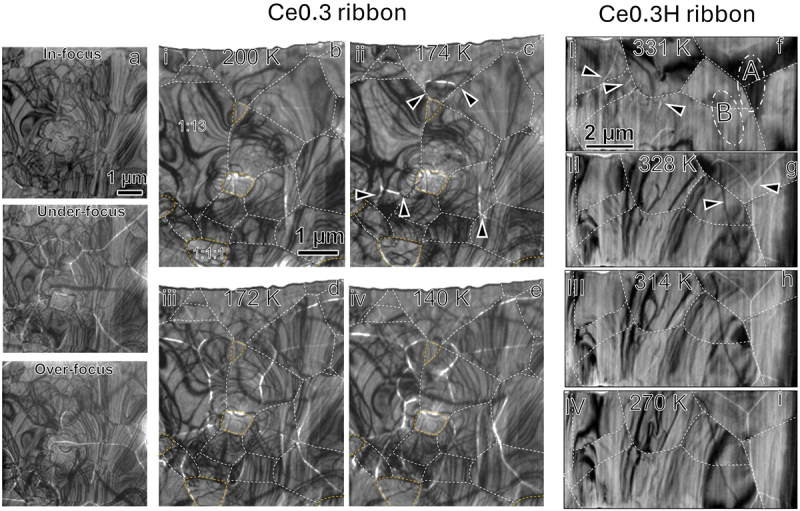
(a) Lorentz TEM Fresnel images obtained at in-focus, under-focus and over-focus states, wherein the magnetic domains show bright contrast in the under-focus state and become dark contrast in over-focus state. Lorentz TEM Fresnel images obtained at various temperatures for (b–e) Ce0.3 ribbon and (f–i) Ce0.3 H ribbon. The white dashed lines indicate the grain boundaries, while the yellow dashed lines mark the 1:1:1 secondary phase, as identified from the bright-field TEM images. The corresponding observation temperatures are labelled as i–iv for the non-hydrogenated sample and I–IV for the hydrogenated sample on the temperature-dependent lattice diagram (Figure 7(b)).

The thermally driven phase transition process was further investigated using Lorentz-TEM in Fresnel mode. Since hydrogenation significantly affects the phase transition, as shown in Figure 2(a-b), we compared the in-situ Lorentz-TEM observations to provide a micro-length scale but direct observation of magneto-structural phase transition in the Ce0.3 ribbon sample and its hydride, as shown in Figure 8. For clarity, the corresponding observation temperatures are marked as i-iv and I-IV for non-hydrogenated and hydrogenated samples on the temperature-dependent lattice diagram (Figure 7(b)), respectively. In the Ce0.3 ribbon sample, which exhibits strong IEM transition Figure 7(b), domain walls (marked by arrowheads) as the temperature approaches the paramagnetic-to-ferromagnetic (PM-FM) phase transition temperature, as shown in Figure 8(b). Note that the magnetic domain walls appear as bright and dark contrast depending on the direction of in-plane magnetization. However, the existence of bend contours makes it difficult to separate the magnetic domain walls with a dark contrast with those of bend contours as an example is provided in Figure 8(a). Hence, for an easy eye guide, we are emphasizing the magnetic domain walls with bright contrast in Figure 8. The magnetic domain walls connect with grain boundaries region, implying that the phase transition preferentially initiates from the triple junctions and does not propagate easily among several grains. With a slight reduction in temperature to approximately 172 K, the number density of domain walls increases substantially and some are crossing the grain boundary region. This indicates that the PM-FM phase transition occurs over a narrow temperature range. Additionally, domain walls emerge not only near grain boundaries but also within grains, likely due to massive phase transformation and the subsequent propagation of ferromagnetic phase into a wider region evidenced by domain walls propagations, which is driven by the temperature change. Upon further cooling to 140 K, no significant changes in the domain wall structure are observed, except for slight displacements of some domain walls within grains. This suggests that the phase transition from PM to FM is nearly complete within this narrow temperature window of 2 K, consistent with the in-situ XRD data shown in Figure 7(b). Figure 8(f) shows a Lorentz-TEM micrograph taken from Ce0.3 hydrogenated ribbon just before the phase transition, where domain walls (marked by arrowheads) begin to form near grain boundaries. Upon further cooling to 328 K, domain walls suddenly appear in regions A and B, accompanied by noticeable contrast changes in these regions. These contrast variations originating from strains can be due to a change in lattice constants during the phase transition, implying the magneto-structure coupling phase transition. Note that the formation of ferromagnetic phase can occur across many grains as shown in Figure 8(g) by marking the magnetic domain walls with bright and dark contrasts. As the temperature is further reduced to 270 K, domain walls exhibit slight displacement within grains, but their overall number density remains unchanged. This suggests that the phase transition from PM to FM is nearly complete at 328 K. Notably, the contrast distribution over the TEM foil sample undergoes negligible change, implying that the strain induced by lattice constant changes upon further cooling below 328 K is negligible, which aligns with the minimal lattice constant change observed between 300 K and 328 K. Lorentz-TEM observations indicate that the nucleation of phase transition occurs consistently at grain boundaries. This suggests that reducing grain size, which increases the volume fraction of grain boundaries, can reduce the energy required for the phase transition. Imperfect structure and possible strains at the grain boundaries facilitates phase nucleation, and reduces energy needed for the transition. This explains why the hysteresis loss decreases from 34 J/kg in coarse-grained Ce0.3 ingot hydrides to 1.7 J/kg in fine-grained Ce0.3 ribbon hydrides. However, this mechanism alone cannot explain the larger hysteresis observed in non-hydrogenated Ce0.3 ribbon samples. This discrepancy may be attributed to the stronger magneto-structural phase transition in non-hydrogenated samples, which overrides the effects of microstructural refinement and results in large hysteresis despite a finer grain structure.

## 4. Conclusion

This study systematically investigates the effects of Ce substitution, grain size refinement via melt spinning, and hydrogenation on the magnetic and magnetocaloric properties of (La,Ce)(Fe,Si)_13_Hₓ alloys. The hydrides obtained from annealed melt-spun ribbons exhibit significantly reduced magnetic hysteresis compared to their ingot counterparts. This reduction is attributed to the finer grain sizes in the ribbons, which increase the grain boundary density. Direct observation of magneto-structural phase transformation using cryogenic Lorentz microscopy reveals that the grain boundaries act as nucleation sites for the phase transition, lowering the associated energy barrier. Hence, increasing the density of grain boundaries as planar defects can be a strategy for reduction of the hysteresis. In-situ XRD measurements further confirm a smaller lattice constant change during the transition, supporting the observed reduced hysteresis. Among the compositions examined, La_0.7_Ce_0.3_(Fe,Si)_13_H_x_ ribbons stand out as a promising candidate for room-temperature magnetic refrigeration. This composition offers a combination of large MCE, reduced cost, low hysteresis, and excellent cyclic stability, as demonstrated by reproducible DSC curves over multiple thermal cycles. In addition, enhanced heat transfer is expected due to the high surface-to-volume ratio in the ribbon form. These features make La_0.7_Ce_0.3_(Fe,Si)_13_H_x_ ribbons attractive for practical applications in room-temperature magnetic cooling systems.

## Supplementary Material

Supplemental Material
