# Supplementary material for: Reduced hysteresis in La0.7Ce0.3Fe11.5Si1.5 hydrides by grain size reduction
Source: Sci Technol Adv Mater. 2025 Jun 30;26(1):2525742. doi: 10.1080/14686996.2025.2525742 (PMC12312218; doi:10.1080/14686996.2025.2525742)
Supplement: Supplemental Material [file TSTA_A_2525742_SM6053.docx]

**Supplementary files**

**Table S1:** Information on atomic positions and site occupancies of Ce0 and Ce0H samples.

|  |  |  | **Ce0** |  |  |  |  | **Ce0H** |  |  |
| --- | --- | --- | --- | --- | --- | --- | --- | --- | --- | --- |
|  | **Atoms** | **x** | **y** | **z** | **Occupancy** | **Atoms** | **x** | **y** | **z** | **Occupancy** |
|  | La1 | 0.25 | 0.25 | 0.25 | 1 | La1 | 0.25 | 0.25 | 0.25 | 1 |
|  | Fe1 | 0 | 0 | 0 | 0.884(6) | Fe1 | 0 | 0 | 0 | 0.884(6) |
| **Ingot** | Si1 | 0 | 0 | 0 | 0.115(4) | Si1 | 0 | 0 | 0 | 0.115(4) |
|  | Fe2 | 0 | 0.1789(9) | 0.1169(4) | 0.884(6) | Fe2 | 0 | 0.1795(3) | 0.1144(2) | 0.884(6) |
|  | Si2 | 0 | 0.1789(9) | 0.1169(4) | 0.115(4) | Si2 | 0 | 0.1795(3) | 0.1144(2) | 0.115(4) |
|  |  |  |  |  |  |  |  |  |  |  |
|  | La1 | 0.25 | 0.25 | 0.25 | 1 | La1 | 0.25 | 0.25 | 0.25 | 1 |
|  | Fe1 | 0 | 0 | 0 | 0.884(5) | Fe1 | 0 | 0 | 0 | 0.884(6) |
| **Ribbon** | Si1 | 0 | 0 | 0 | 0.115(1) | Si1 | 0 | 0 | 0 | 0.115(4) |
|  | Fe2 | 0 | 0.179(1) | 0.116(9) | 0.89 | Fe2 | 0 | 0.1795(3) | 0.1144(2) | 0.884(6) |
|  | Si2 | 0 | 0.179(1) | 0.116(9) | 0.11 | Si2 | 0 | 0.1795(3) | 0.1144(2) | 0.115(4) |

**Table S2:** Information on atomic positions and site occupancies of Ce0.3 and Ce0.3H samples.

|  |  |  | **Ce0.3** |  |  |  |  | **Ce0.3H** |  |  |
| --- | --- | --- | --- | --- | --- | --- | --- | --- | --- | --- |
|  | **Atoms** | **x** | **y** | **z** | **Occupancy** | **Atoms** | **x** | **y** | **z** | **Occupancy** |
|  | La1 | 0.25 | 0.25 | 0.25 | 0.70(6) | La1 | 0.25 | 0.25 | 0.25 | 0.70(6) |
|  | Ce1 | 0.25 | 0.25 | 0.25 | 0.30(6) | Ce1 | 0.25 | 0.25 | 0.25 | 0.30(6) |
|  | Fe1 | 0 | 0 | 0 | 0.884(6) | Fe1 | 0 | 0 | 0 | 0.884(6) |
| **Ingot** | Si1 | 0 | 0 | 0 | 0.115(4) | Si1 | 0 | 0 | 0 | 0.115(4) |
|  | Fe2 | 0 | 0.1789(9) | 0.1169(4) | 0.884(6) | Fe2 | 0 | 0.1795(3) | 0.1144(2) | 0.884(6) |
|  | Si2 | 0 | 0.1789(9) | 0.119(4) | 0.115(4) | Si2 | 0 | 0.1795(3) | 0.1144(2) | 0.115(4) |
|  |  |  |  |  |  |  |  |  |  |  |
|  | La1 | 0.25 | 0.25 | 0.25 | 0.70(5) | La1 | 0.25 | 0.25 | 0.25 | 0.70(4) |
|  | Ce1 | 0.25 | 0.25 | 0.25 | 0.30(4) | Ce1 | 0.25 | 0.25 | 0.25 | 0.30(5) |
|  | Fe1 | 0 | 0 | 0 | 0.884(6) | Fe1 | 0 | 0 | 0 | 0.884(6) |
| **Ribbon** | Si1 | 0 | 0 | 0 | 0.115(4) | Si1 | 0 | 0 | 0 | 0.140(8) |
|  | Fe2 | 0 | 0.1789(9) | 0.1169(4) | 0.884(6) | Fe2 | 0 | 0.179(1) | 0.115(2) | 0.859(2) |
|  | Si2 | 0 | 0.1789(9) | 0.1169(4) | 0.115(4) | Si2 | 0 | 0.1795(3) | 0.1144(2) | 0.115(4) |

**Table S3:** Refinement parameters for the sample studied this work.

| Sample | R_Wp_ | ${}_{reduced}^{2}$ |
| --- | --- | --- |
| Ce0 ingot | 8.9 | 9.4 |
| Ce0.3 ingot | 10.9 | 9.8 |
| Ce0 ribbon | 9 | 9.6 |
| Ce0.3 ribbon | 7.9 | 8 |
| Ce0H ingot | 9.9 | 9.5 |
| Ce0.3H ingot | 9.2 | 9.7 |
| Ce0H ribbon | 7.6 | 5.7 |
| Ce0.3H ribbon | 8 | 7.4 |

**
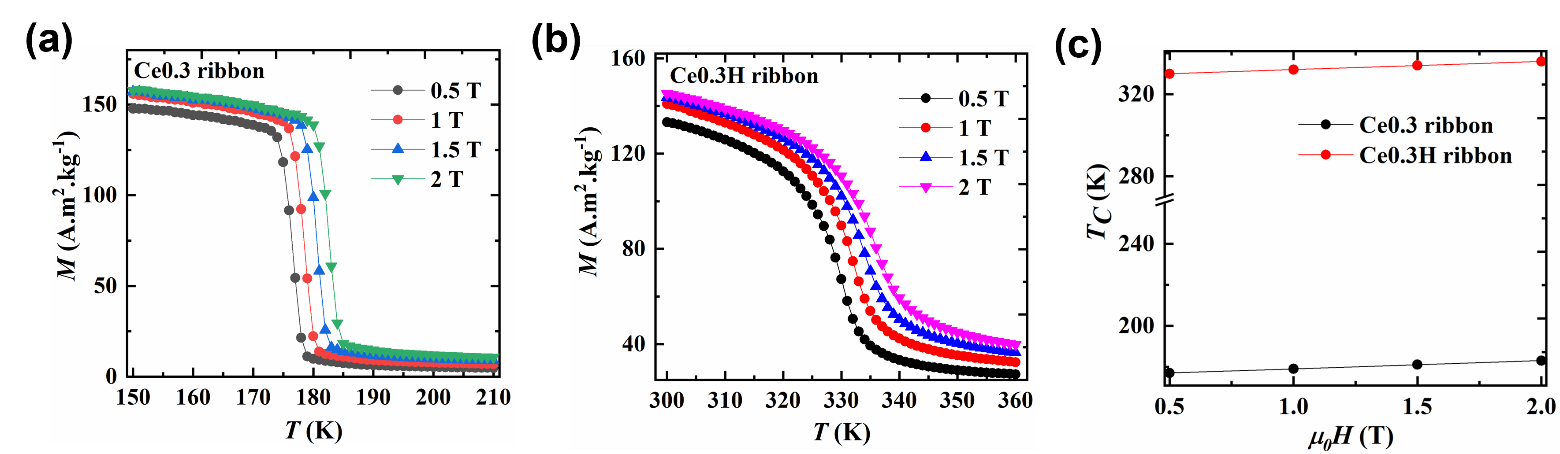
**

**Fig. S1:** M-T curves of (a) Ce0.3 ribbon and (b) Ce0.3H ribbon at different magnetic fields and (c) Transition temperature against magnetic field for Ce0.3 ribbon and Ce0.3H ribbon.

**
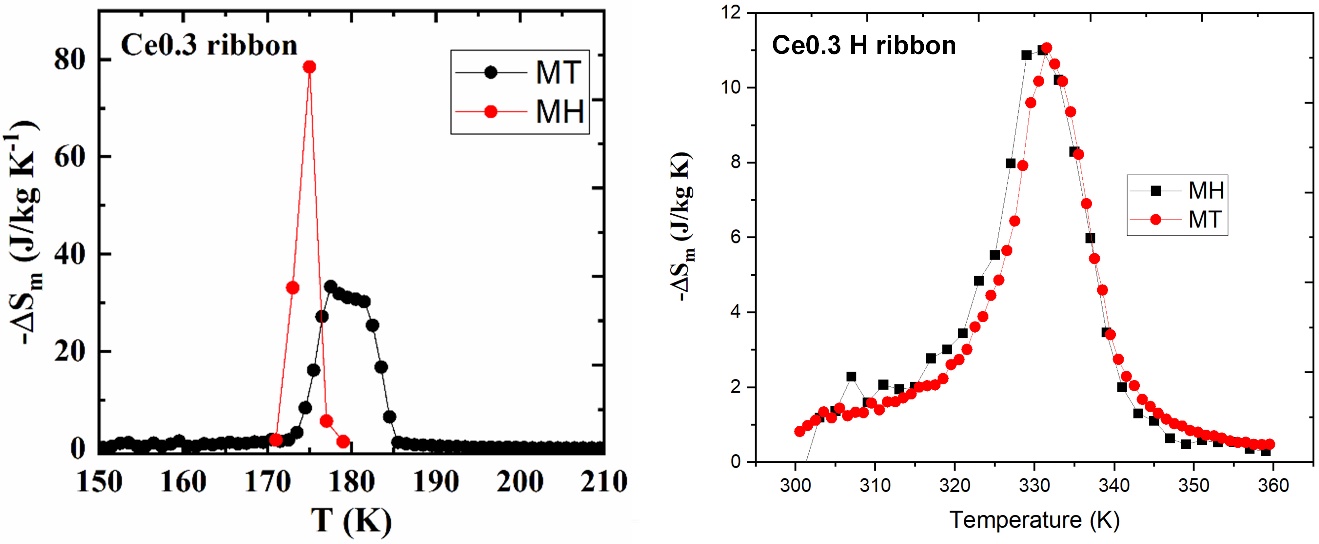
**

**Fig. S2:**  Comparison of **-**ΔS_m_ obtained from isothermal (MH) as well as isofield (MT) data for Ce0.3H ribbon.





**Figure S3:** The magnetic entropy and magnetic hysteresis of this work compared with reported data of La_0.7_(Ce,Pr,Nd)_0.3_Fe_11.6_Si_1.4_H_1.6_ [1], LaFe_11.7_Si_1.3_C_0.2_H_x_ [2], (La_0.6_Ce_0.4_)_2_Fe_11_SiH_y_ [3], La_0.5_Pr_0.5_Fe_11.4_Si_1.6_H_0.9_ [4], La_0.5_Pr_0.5_Fe_11.4_Si_1.6_H_1.6_ [4], La_0.5_Pr_0.5_Fe_11.4_Si_1.6_H_1.6_ [5], La_0.7_ Pr_0.3_ Fe_11.5_Si_1.5_ C_0.2_ H_0.6_ [6], La_0.7_ Pr_0.3_Fe_11.5_Si_1.5_ C_0.2_ H_1.2_ [6], La_0.8_Ce_0.2_(Fe_0.985_Mn_0.015_)_11.5_Si_1.5_H_y_ [7], La_0.8_Ce_0.2_(Fe_0.979_Mn_0.021_)_11.5_Si_1.5_H_y_ [7], La_0.8_Ce_0.2_(Fe_0.975_Mn_0.025_)_11.5_Si_1.5_H_y_ [7] and LaFe_11.7_Si_1.3_C_0.2_H_1.7_ [8].

[1]. Bao LF, Hu FX, Chen L, et al. Magnetocaloric properties of La(Fe,Si)_13_-based material and its hydride prepared by industrial mischmetal. Appl. Phys. Lett. 2012;101:162406. doi: <https://doi.org/10.1063/1.4760262>

[2]. Zhang H, Long Y, Niu E, et al. Influence of particle size on the hydrogenation in La(Fe, Si)_13_ compounds. J. Appl. Phys. 2013;113:17A911. doi: <https://doi.org/10.1063/1.4794975>

[3]. Liu Y, Fu X, Yu Q, et al. Significant reduction of phase-transition hysteresis for magnetocaloric (La_1-_*_x_*Ce*_x_*)_2_Fe_11_Si_2_H*_y_* alloys by microstructural manipulation. Acta Mater. 2021;207:116687. doi: <https://doi.org/10.1016/j.actamat.2021.116687>

[4]. Zhao JL, Shen J, Hu FX, et al. Reduction of magnetic hysteresis loss in La_0.5_Pr_0.5_Fe_11.4_Si_1.6_H_x_ hydrides with large magnetocaloric effects. J. Appl. Phys. 2010;107:113911. doi:10.1063/1.3374635

[5]. Debnath JC, Zeng R, Kim JH, et al. Appl. Phys. A. Reduction of hysteresis loss in LaFe_11.7_ Si_1.3_ H_x_ hydrides with significant magnetocaloric effects. 2012;106:245. doi: 10.1007/s00339-011-6571-1

[6]. Zhao JL, Shen J, Zhang H, et al. Hydrogenating process and magnetocaloric effect in La_0.7_Pr_0.3_Fe_11.5_Si_1.5_C_0.2_H_x_ hydrides. J. Alloys Compd. 2012;520:277-280. doi: 10.1016/j.jallcom.2012.01.042

[7]. Wang C, Long Y, Ma T, et al. The hydrogen absorption properties and magnetocaloric effect of La_0.8_Ce_0.2_(Fe_1−x_Mn_x_)_11.5_Si_1.5_H_y_. J. Appl. Phys. 2011;109:07A910. doi: <https://doi.org/10.1063/1.3549560>

[8]. Zhang H, Shen BG, Xu ZY, et al. Reduction of hysteresis loss and large magnetocaloric effect in the C- and H-doped La(Fe, Si)_13_ compounds around room temperature. J. Appl. Phys. 2012;111:07A909. doi: <https://doi.org/10.1063/1.3670608>


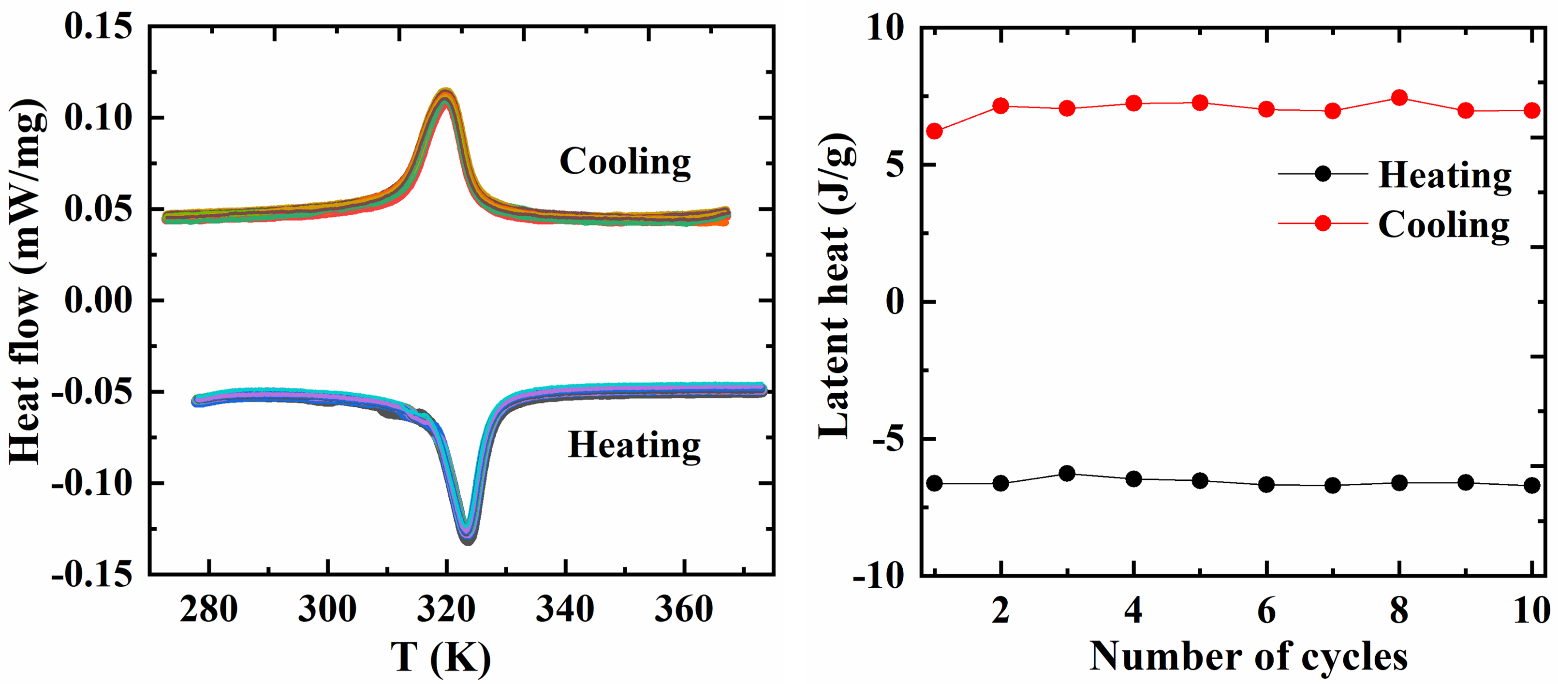


**Figure S4:** DSC curves of 10 cycles and the latent heat for each cycle of Ce0.3H ribbon.


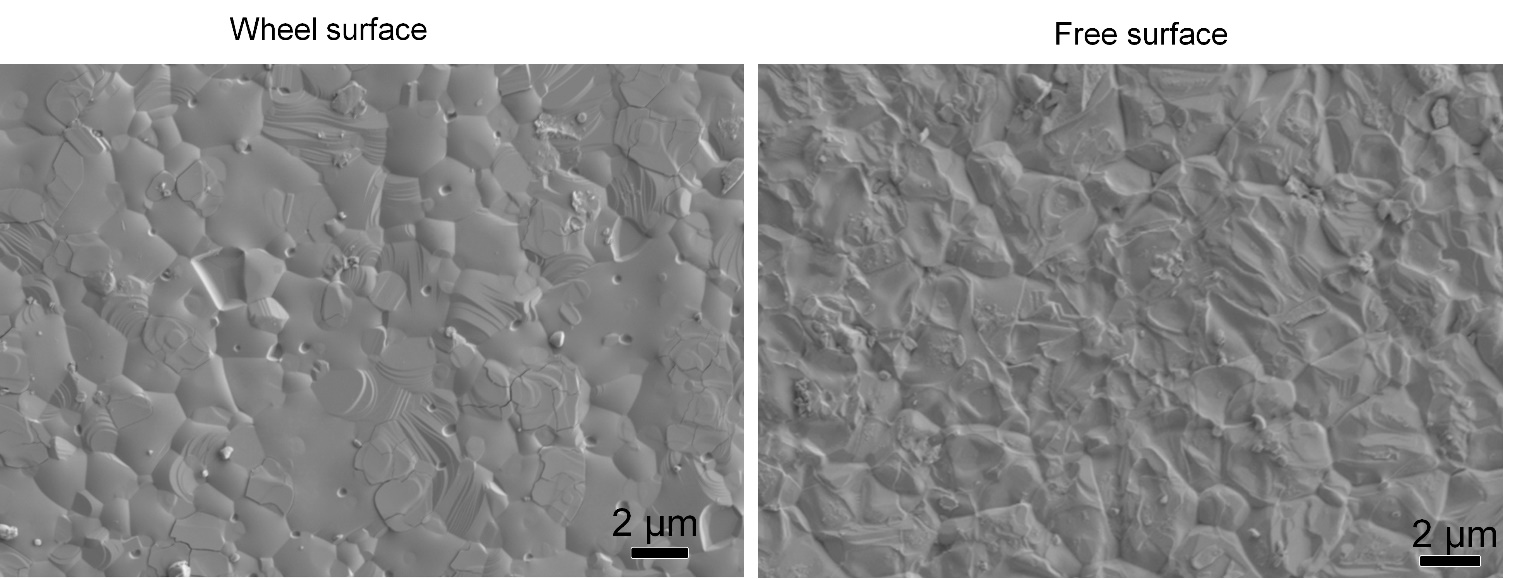


**Figure S5:** Secondary electron (SE)-SEM image of wheel surface and free surface of Ce0.3H ribbon.


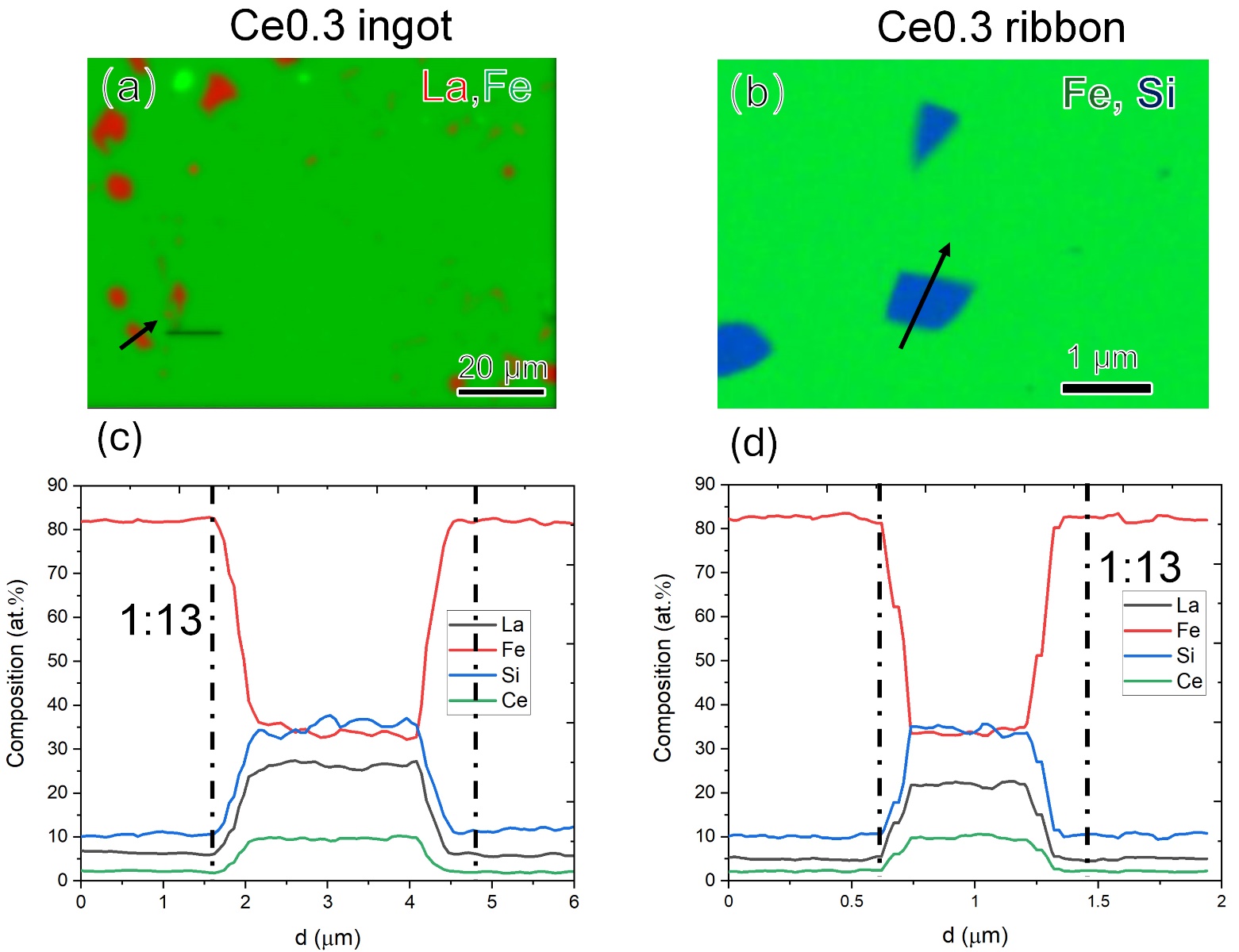


**Figure S6:** Compositional profiles obtained across secondary phase marked by arrowheads in (a-b) for Ce0.3 ingot and Ce0.3 ribbon, respectively. The corresponding EDS compositional profile for (c) Ce0.3 ingot and (d) Ce0.3 ribbon.





**Figure S7:** Temperature dependence of the exponent n for Ce0.3H ingot and Ce0.3H ribbon.
